# Association between serum copper and blood glucose: a mediation analysis of inflammation indicators in the NHANES (2011–2016)

**DOI:** 10.3389/fpubh.2024.1401347

**Published:** 2024-05-24

**Authors:** Zijing Cheng, Yuzhe Kong, Wenqi Yang, Haitao Xu, Decheng Tang, Yu Zuo

**Affiliations:** ^1^Xiangya School of Medicine, Central South University, Changsha, Hunan, China; ^2^Department of Management Science, School of Management, Fudan University, Shanghai, China; ^3^Third Xiangya Hospital, Central South University, Changsha, China

**Keywords:** serum copper, inflammatory factor, blood glucose, National Health and Nutrition Examination Survey, mediation analysis

## Abstract

**Background:**

The rising prevalence of diabetes underscores the need for identifying effective prevention strategies. Recent research suggests environmental factors, particularly heavy metals like copper, significantly influence health outcomes, including diabetes, through mechanisms involving inflammation and oxidative stress. This study aims to explore how serum copper levels affect blood glucose, employing NHANES data from 2011 to 2016, to provide insights into environmental health’s role in diabetes prevention and management.

**Methods:**

The study analyzed data from 2,318 NHANES participants across three cycles (2011–2016), focusing on those with available data on serum copper, inflammatory markers, and blood glucose levels. We utilized principal component analysis for selecting inflammatory markers, mediation analysis to examine direct and indirect effects, multiple linear regression for assessing relationships between markers and glucose levels, and weighted quantile sum regression for evaluating individual and collective marker effects, adjusting for demographic variables and serum copper.

**Results:**

Participants averaged 42.70 years of age, with a near-even split between genders. Average serum copper was 119.50 μg/dL, white blood cell count 6.82 × 109/L, and fasting blood glucose 107.10 mg/dL. Analyses identified significant mediation by inflammatory markers (especially white blood cells: 39.78%) in the copper-blood glucose relationship. Regression analyses highlighted a positive correlation between white blood cells (estimate: 1.077, 95% CI: 0.432 to 2.490, *p* = 0.013) and copper levels and a negative correlation for monocyte percentage (estimate: −1.573, 95% CI: 0.520 to −3.025, *p* = 0.003). Neutrophil percentage was notably influential in glucose levels. Sensitive analyses confirmed the study’s findings.

**Conclusion:**

Serum copper levels significantly impact blood glucose through inflammatory marker mediation, highlighting the importance of considering environmental factors in diabetes management and prevention. These findings advocate for public health interventions and policies targeting environmental monitoring and heavy metal exposure reduction, emphasizing the potential of environmental health measures in combating diabetes incidence.

## 1 Introduction

High blood glucose, indicative of diabetes, leads to a significant and often irreversible decline in health, contributing to cardiovascular diseases, kidney failure, and vision loss (1–4). It represents a major cause of disability worldwide, with a growing prevalence especially alarming among the older adult (5). These highlight the urgency in identifying effective primary prevention measures for managing high blood glucose (BG) levels (6, 7).

One of the myriad factors influencing BG levels is the concentration of heavy metals in serum. Beyond dietary influences, the prevalence of diabetes mellitus is notably exacerbated by exposure to various environmental contaminants, including heavy metals, whose presence in the atmosphere has surged alongside industrialization (8–10). Research indicates a substantial positive correlation between the concentration of heavy metals in the blood and their atmospheric levels, suggesting that the accumulation of specific heavy metals in the human body impacts BG concentration (11). For instance, cadmium (Cd) accumulation in insulin-producing β-cells can diminish insulin release and elevate BG levels, while lead (Pb) has been linked to increased insulin resistance and a higher risk of diabetes mellitus (12, 13).

Inflammation also plays a critical role in regulating BG levels. Insights from the Framingham Offspring Study reveal a direct correlation between insulin resistance and elevated markers of oxidative stress. This oxidative stress compromises the ability of muscle and adipose tissues to absorb glucose, as well as impairing pancreatic islet cells’ insulin secretion capacity, thereby contributing to higher BG concentrations (14). Reactive oxygen species generated within the body inflict damage on cellular DNA, membranes, lipids, and proteins, further inducing the expression of inflammatory genes. Such inflammation disrupts insulin-mediated metabolic pathways, culminating in insulin resistance (15).

Serum heavy metal concentrations are mainly related to the external environment, with heavy metals entering the body through inhalation, ingestion, and dermal contact, while inflammation and oxidative stress are usually caused by other *in vivo* abnormalities (16). Relevant studies have shown that heavy metal exposure induces systemic inflammatory responses, and disruption of metal ion homeostasis can also lead to oxidative stress, for example, Cu induces oxidative stress through two pathways, namely, catalyzing the formation of ROS via a related reaction as well as decreasing glutathione levels, and zinc deficiency increase oxidative damage levels to some degree, thus it is envisioned that serum heavy metals affect BG levels as being mediated by inflammation and oxidative stress *in vivo* (16–18).

However, although existing researches have highlighted the roles of diet and genetic factors in the onset of diabetes, in-depth studies into the impact of environmental factors, particularly heavy metals, on diabetes were scarce. As environmental exposure to heavy metals such as Cd has been confirmed to increase the risk of diabetes, there still remains a significant research gap regarding how copper, a common environmental metal, affects blood glucose levels through internal biological processes. Moreover, although inflammation was widely considered key pathways in the progression of diabetes, systematic studies on how these pathways mediate the interaction between copper and blood glucose levels are extremely limited. Current research tends to focus on individual biomarkers, overlooking the complexities of the combined effects of multiple markers.

Therefore, the present study was to analyze the correlation between Cu and the concentration of BG and the mediating role of inflammatory factors therein by means of a large-scale cross-sectional study based on the NHANES database.

## 2 Materials and methods

### 2.1 Study population

Led by the National Center for Health Statistics (NCHS) at the Centers for Disease Control and Prevention (CDC), NHANES is a biannual program of studies designed to assess the health and nutritional status of adults and children in the United States. NHANES is designed as a multiyear, stratified, clustered four-stage sample of non-institutionalized civilians with fixed sample-size targets for sampling domains defined by age, sex, race and ethnicity, and socioeconomic status, with data released in 2-y cycles.

Participants gave informed consent of the survey process and their rights as a participant, and the survey was approved by the NCHS Review Board.27 Questionnaires were administered in-home followed by standardized health examinations in specially equipped mobile examination centers. Publicly available, de-identified, and detailed health data sets are available on the NHANES website.^1^ We acquired all data from the NHANES database that measured Cu levels in serum, including 3 2-y cycles (2011–2016) and all of the cycles with available data on blood and urinary metals and detailed drug use, to create a larger and more geographically diverse sample.

### 2.2 Exclusion criteria

**Figure 1:**
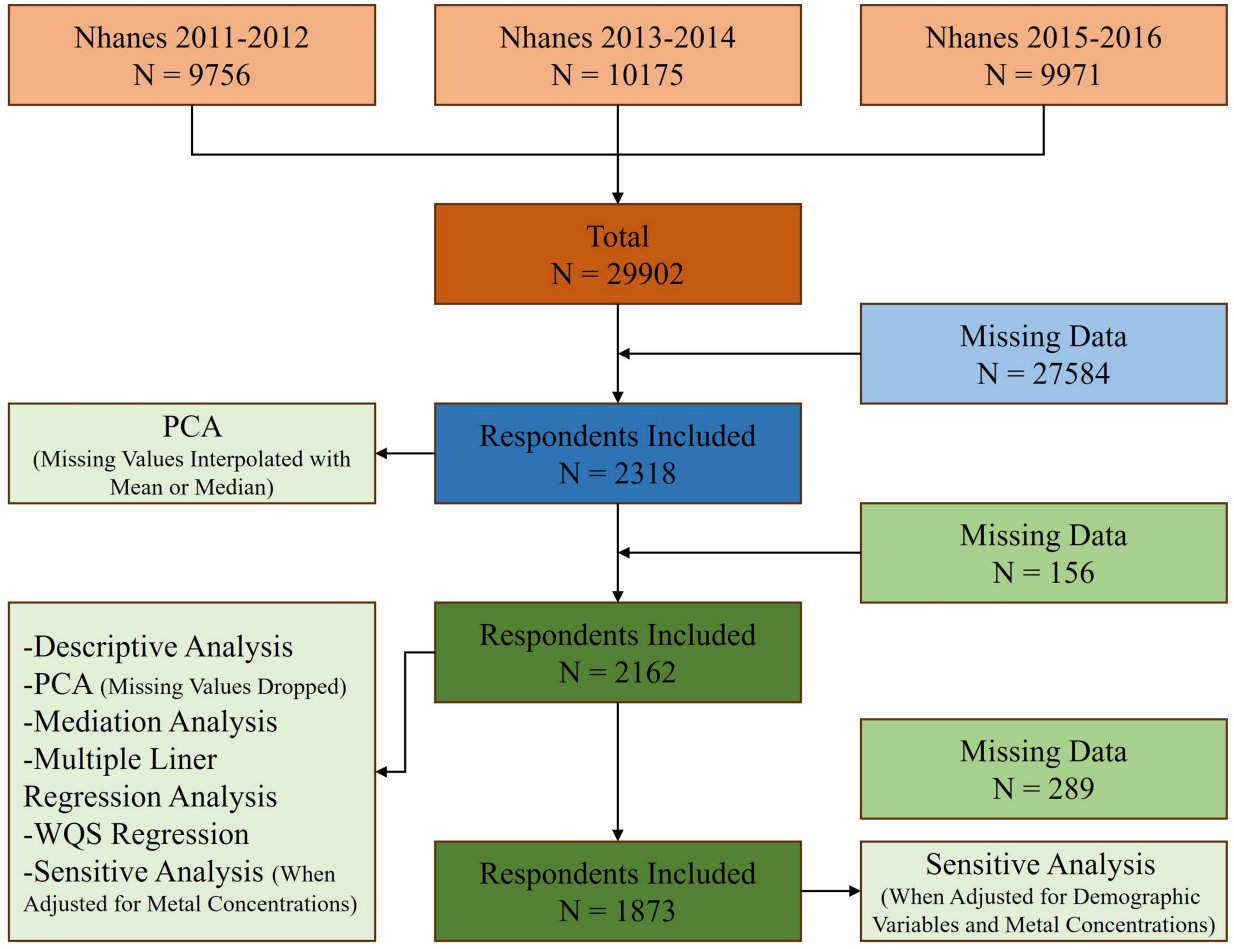
Study flowchart.

Data from 3 cycles of NHANES from 2011 to 2016 were used in this study. First, a total of 29,902 individuals participated in the cross-sectional study. After excluding participants without serum copper concentration, inflammatory markers, and BG concentration data types, 2,318 participants were included in this study. In addition to performing PCA by substituting missing values using mean or median for missing values, we excluded participants with missing data, and 2,162 participants were included in subsequent statistical analyses. Finally, for sensitivity analyses (when adjusted for demographic variables and metal concentrations), we excluded participants with missing data on demographic variables, and a total of 1873 participants were included (Figure 1).

The NHANES database was approved by the National Center for Health Statistics and was directly accessible to researchers who met eligibility requirements. All participants in the database signed an informed consent form.

### 2.3 Measurement of serum Cu

Serum specimens were processed and stored under appropriate frozen (−70°C) conditions until they were shipped to the National Center for Environmental Health for analysis. Serum Cu concentrations were measured by inductively coupled plasma dynamic reaction cell mass spectrometry (ICP-DRC-MS)—a multi-element analytical technique capable of trace-level elemental analysis. Liquid samples were introduced into the ICP through a nebulizer and spray chamber carried by a flowing argon stream. Radio-frequency power was coupled into flowing argon to form a plasma. The sample passed through a region of the plasma, and the thermal energy atomized the sample and then ionized the atoms. The ions, along with the argon, entered the mass spectrometer through an interface that separated the ICP from the mass spectrometer. The ions passed through a focusing region, dynamic reaction cell, and the quadrupole mass filter, and finally, were counted in rapid sequence at the detector allowing individual isotopes of an element to be determined. The isotopes measured by this method included Zn (m/z 64), Cu (m/z 65), and Se (m/z 78), as well as the internal standard, gallium (m/z 71). Serum samples were diluted 1 + 1 + 28 with water and diluent containing gallium (Ga) for multi-internal standardization.

### 2.4 Measurement of inflammation biomarkers

Fasting blood sample of the participants for the laboratory tests was collected by the mobile examination center phlebotomist. The NHANES laboratory manual provides the reference ranges on laboratory parameters in the form of lower and upper limits. Analysis for the complete blood count was done in the mobile examination center, and refrigerated or frozen blood samples were transported and analyzed in the central laboratories for the other parameters.

The DxC800 with lactate dehydrogenase (LDH) reagent (using lactate as substrate) utilizes an enzymatic rate method to measure LDH activity in biological fluids. The DxC600i system or DxC800 system uses a kinetic rate method using a 2-Amino-2-Methyl-1-Propanol (AMP) buffer to measure alkaline phosphatase (ALP) activity in serum or plasma. For the processing of CRP, latex-enhanced nephelometry with particle-enhanced assays was used for quantitation. These assays were performed on a Behring Nephelometer for quantitative CRP determination. The methods used to derive complete blood count (CBC) parameters [white blood cell (WBC), segmented neutrophils (Nsg), lymphocyte (Lym), monocyte count (Mono), eosinophils (Eos) and basophils (Baso)] are based on the Beckman Coulter method of counting and sizing, in combination with an automatic diluting and mixing device for sample processing. The Beckman Coulter MAXM instrument in the Mobile Examination Centers (MECs) produces a CBC on blood specimens.

### 2.5 Measurement of BG

The method for measuring BG levels involves using the Roche Cobas C311 system, which first requires the patient to be fasting or to undergo an oral glucose tolerance test. Blood is collected via venipuncture, and plasma samples are collected in fluoride-containing gray top tubes, with a minimum volume of 200 microliters. To reduce sugar decomposition, the collected samples must be immediately placed in an ice water bath, and plasma and cells must be separated within 30 min. If testing is not conducted immediately, the samples are then frozen and stored at −70°C. The measurement principle is based on the reaction catalyzed by hexokinase between glucose and ATP to produce glucose-6-phosphate (G-6-P) and ADP, then G-6-P is further oxidized by glucose-6-phosphate dehydrogenase, simultaneously generating NADH directly proportional to the glucose concentration, which is measured by spectrophotometry at 340 nanometers. System calibration and quality control are performed before testing to ensure the accuracy and repeatability of the results. The Roche Cobas C311 automatically calculates the glucose concentration, and the results are reported in mg/dL or mmol/L.

### 2.6 Covariates

Age, sex, race and ethnicity, education, marriage, and PIR were acquired from self-reported questionnaires on demographic information. Race and ethnicity are classified as Mexican American, Other Hispanic, Non-Hispanic White, Non-Hispanic Black, and Other Race (including Multiple Races) based on self-identification originally designated by NHANES. “Other Race” encompasses all other races, including Non-Hispanic Asians and individuals reporting multiple races. Common exposures to other metals (e.g., dietary) may vary by race and ethnicity, hence are adjusted for in our models. Education is reclassified into Less than 9th grade, 9–11th grade (Includes 12th grade with no diploma), High school graduate/GED or equivalent, Some college or AA degree, and College graduate or above. Marital status is divided into married, widowed, divorced, separated, never married, living with partner, refused, and missing.

### 2.7 Statistical analysis

First, we utilized PCA to determine the combination of inflammatory markers adopted in this study. Second, we conducted a descriptive analysis of the serum copper (Cu), inflammatory markers, and BG concentration in the included population, reporting mean, standard deviation, median, and quartiles.

Third, we performed mediation analysis to explore the direct and indirect relationships and the extent of mediation effect using non-parametric bootstrapping (*n* = 1,000).

Fourth, we conducted linear regression analysis to investigate the mixed effects of different inflammatory markers on glucose concentration. Fifth, we performed WQS regression analysis to assess the comprehensive and individual effects of inflammatory markers on BG concentration by calculating weighted linear indices and assigning corresponding weights. In this study, 10,000 bootstrap iterations were used to construct positive and negative WQS indices. When the WQS index was significant, the corresponding weights were examined to determine the relative contribution of each heavy metal in the index to glucose concentration. The dataset was randomly split, with 40% allocated to the training set and the remaining 60% as the validation set.

Finally, we conducted the sensitivity analysis. We adjusted for demographic variables in mediation analyses and for serum copper concentrations as well as demographic variables in MLRA, WQS regression analyses. We also performed sensitivity analyses of PCA using different missing value treatments to determine the optimal combination of inflammatory indicators for inclusion in the study.

Adjusted for demographic factors, weighted data was not used. All analyses, including WQS, MLRA (Multiple Linear Regression Analysis), and mediation analysis, were conducted using R software. A *p*-value of <0.05 was considered statistically significant.

## 3 Results

### 3.1 General information

This study involved 2,318 participants (Table 1), evenly split between males (50.17%, *n* = 1,163) and females (49.83%, *n* = 1,155), with an average age of 42.70 years (*SD* = 15.34). The population was diverse, including Mexican American (14.41%), other Hispanic (12.21%), non-Hispanic White (33.69%), non-Hispanic Black (22.30%), and other races (17.39%). Educational levels ranged from less than 9th grade (8.02%) to college graduate or above (26.92%). Marital status varied, with 47.63% married and 21.05% never married. The median poverty income ratio (PIR) was 1.94 (IQR = 0.97–3.81).

**Table 1:** Baseline characteristics of participants included in the study.

Item		Data		
		*n* (Mean)	% (*SD*)	Median (Q1, Q3)
Population		2,318	/	/
Gender	Male	1,163	50.17	/
	Female	1,155	49.83	/
Age		42.70	15.34	42.00 (29.00, 56.00)
Race	Mexican American	334	14.41	/
	Other Hispanic	283	12.21	/
	Non-Hispanic White	781	33.69	/
	Non-Hispanic Black	517	22.30	/
	Other Race – Including Multi-Racial	403	17.39	/
Education level	Less than 9th grade	186	8.02	/
	9–11th grade (Includes 12th grade with no diploma)	332	14.32	/
	High school graduate/GED or equivalent	512	22.09	/
	Some college or AA degree	664	28.65	/
	College graduate or above	624	26.92	/
	Missing	0	0.00	/
Marital status	Married	1,104	47.63	/
	Widowed	64	2.76	/
	Divorced	231	9.97	/
	Separated	69	2.98	/
	Never married	488	21.05	/
	Living with partner	206	8.89	/
	Refused	2	0.09	/
	Missing	154	6.64	/
PIR		2.37	1.64	1.94 (0.97, 3.81)

PIR stands for “People Income Ratio”.

### 3.2 Principal component analysis

First, we conducted a principal component analysis (PCA) to explore the combination of inflammatory markers in the study. We calculated the standard deviation (S.D.) and explained variance (VER) for each marker, resulting in the cumulative explained variance (CVER) across the components.

After imputing missing values with the mean, the analysis indicated that WBC count, Lym percentage, Mono percentage, Nsg percentage, Eos percentage and Baso percentage exhibited higher variances, with their cumulative variance accounting for a substantial majority of data variability (Table 2). Similarly, imputing missing values with the median or removing missing values altogether yielded comparable results.

**Table 2:** Factor loadings of 11 inflammation indicators based on principal component analysis.

	WBC	LymPCT	MonoPCT	NsgPCT	EosPCT	BasoPCT	LymC	MonoC	NsgC	EosC	BasoC
Model I											
S.D.	1.8627	1.5654	1.3369	1.2126	1.2050	0.4465	0.2698	0.2377	0.2028	0.0230	0.0062
VER	0.3154	0.2228	0.1625	0.1337	0.1320	0.0181	0.0066	0.0051	0.0037	0.0001	0.0000
CVER	0.3154	0.5382	0.7007	0.8343	0.9663	0.9845	0.9911	0.9962	1.0000	1.0000	1.0000
Model II											
S.D.	1.8618	1.5671	1.3359	1.2086	1.2047	0.4540	0.2757	0.2377	0.2035	0.0310	0.0147
VER	0.3151	0.2233	0.1622	0.1328	0.1319	0.0187	0.0069	0.0051	0.0038	0.0001	0.0000
CVER	0.3151	0.5384	0.7006	0.8334	0.9653	0.9841	0.9910	0.9961	0.9999	1.0000	1.0000
Model III											
S.D.	1.8627	1.5654	1.3369	1.2126	1.2050	0.4465	0.2697	0.2377	0.2028	0.0204	0.0062
VER	0.3154	0.2228	0.1625	0.1337	0.1320	0.0181	0.0066	0.0051	0.0037	0.0000	0.0000
CVER	0.3154	0.5382	0.7007	0.8343	0.9663	0.9845	0.9911	0.9962	1.0000	1.0000	1.0000

LymPCT, Lym percentage; MonoPCT, mono percentage; NsgPCT, Nsg percentage; EosPCT, Eos percentage; BasoPCT, Baso percentage; LymC, Lym count; MonoC, Mono count; NsgC, Nsg count; EosC, Eos count; BasoC, Baso count. Model I used the overall mean to fill in the missing values, Model II used the overall median to fill in the missing values, and Model III removed the missing values.

### 3.3 Level of serum Cu, inflammation markers and BG

Then, we analyzed the baseline levels for serum Cu, WBC count, Lym percentage, Mono percentage, Nsg percentage, Eos percentage, Baso percentage, and BG (Table 3). The mean serum Cu level was found to be 119.50 μg/dL, with a standard deviation (SD) of 32.04, highlighting a moderate variability among individuals. WBC count averaged at 6.82 × 10^9^/L. Lym percentage, Mono percentage, Nsg percentage, Eos percentage, and Baso percentage demonstrated diverse immune cell distribution, with mean values of 31.67, 7.89, 56.77, 2.98, and 0.76%, respectively. The mean BG level was noted at 107.10 mg/dL (*SD* = 36.62).

**Table 3:** Level of serum Cu, inflammation markers and blood glucose among the participants included in the study.

	Mean	*SD*	Median	Q1	Q3		Mean	*SD*	Median	Q1	Q3
Serum Cu	119.50	32.04	113.80	98.00	133.98	NsgPCT	56.77	9.46	57.10	50.50	63.30
WBC	6.82	2.05	6.50	5.40	7.90	EosPCT	2.98	2.21	2.40	1.60	3.70
LymPCT	31.67	8.52	31.20	25.80	36.80	BasoPCT	0.76	0.45	0.70	0.50	0.90
MonoPCT	7.89	2.20	7.60	6.40	9.10	BG	107.10	36.62	99.00	92.00	108.00

LymPCT, Lym percentage; MonoPCT, Mono percentage; NsgPCT, Nsg percentage; EosPCT, Eos percentage; BasoPCT, Baso percentage.

### 3.4 Mediation analysis

Then, we conducted the mediation analysis investigating the role of inflammatory factors in the relationship between serum Cu levels and BG, we focused on four intermediary variables: WBC count, Lym percentage, Mono percentage, Nsg percentage (Table 4). The analysis revealed significant mediation effects, with WBC count showing a substantial mediation proportion of 39.78% (*p* = 0.0004), indicating a strong mediator role. Lym percentage displayed a moderate mediation effect with a proportion of 10.16% (*p* = 0.0421), while Mono percentage also demonstrated significant mediation, with a proportion of 33.37% (*p* = 0.0006). Nsg percentage contributed notably as well, with a mediation proportion of 20.82% (*p* = 0.0076).

**Table 4:** The mediating effects of the relationship between serum Cu and BG.

Intermediary Variable	Indirect effects β (95% CI)			Direct effects β (95% CI)			Total effects β (95% CI)			Mediated proportion	*p*-value
	Estimate	CI lower	CI upper	Estimate	CI lower	CI upper	Estimate	CI lower	CI upper		
WBC	0.0234	−0.0193	0.0661	0.0155	0.0069	0.0240	0.0389	−0.0032	0.0810	0.3978	0.0004
LymPCT	0.0350	−0.0072	0.0771	0.0040	0.0001	0.0078	0.0389	−0.0032	0.0810	0.1016	0.0421
MonoPCT	0.0259	−0.0166	0.0684	0.0130	0.0055	0.0204	0.0389	−0.0032	0.0810	0.3337	0.0006
NsgPCT	0.0308	−0.0116	0.0732	0.0081	0.0022	0.0141	0.0389	−0.0032	0.0810	0.2082	0.0076

LymPCT, Lym percentage; MonoPCT, Mono percentage; NsgPCT, Nsg percentage.

### 3.5 Multiple linger regression analysis

Building on the results from the mediation analysis, we further investigated the influence of significant mediatory variables on the relationship between serum Cu levels and BG through multiple linear regression analysis.

The WBC presented a positive and significant association with serum Cu (estimate: 1.077, 95% CI: 0.432 to 2.490, *p* = 0.013), suggesting higher WBC counts correspond with increased serum Cu levels. Mono showed a notable negative relationship (estimate: −1.573, 95% CI: 0.520 to −3.025, *p* = 0.003), indicating that higher Mono is associated with decreased serum Cu levels. Lym and Nsg did not demonstrate statistically significant associations (*p*-values of 0.126 and 0.210, respectively) (Table 5).

**Table 5:** Association between potential mediators and BG determined by MLRA.

Variable	Unadjusted model				Variable	Unadjusted model			
	Estimate	Lower CI	Upper CI	*p-*value		Estimate	Lower CI	Upper CI	*p-*value
WBC	1.0769	0.4325	2.4902	0.0128	MonoPCT	−1.5743	−2.5988	−0.5498	0.0026
LymPCT	−0.5403	0.3529	−1.5309	0.1259	NsgPCT	−0.4527	−1.1208	0.2154	0.1840

LymPCT, Lym percentage; MonoPCT, Mono percentage; NsgPCT, Nsg percentage.

### 3.6 WQS regression models

Subsequently, we employed WQS regression analysis to further explore the weighted impact of inflammatory factors on BG. The WQS regression results showed that the overall model estimate was 4.7167, with statistical significance (*p*-value = 0.0216), indicating a significant effect of the combination of inflammatory factors on BG. Among the inflammatory factors, Nsg had the highest weight coefficient (0.4472). WBC and Lym had weight coefficients of 0.3558 and 0.1954, respectively. Meanwhile, the weight coefficient for Mono was 0.0015, indicating its relatively minor impact on BG.

### 3.7 Sensitive analysis

We conducted several sensitivity analyses to verify the stability of our research results. Initially, after adjusting for demographic variables in the mediation analysis, the results showed a significant increase in the mediation effects of WBC and Mono (*p* < 0.005), while the mediation effect of Lym became non-significant (*p* = 0.7203) (Supplementary Table S1). Secondly, upon adjusting for serum Cu levels and then for serum Cu levels plus demographic variables in the MLRA, the negative correlation between Mono and serum Cu levels remained significant across all models (*P* < =0.003), whereas the correlation of WBC became non-significant after including demographic variables (*p* = 0.072) (Supplementary Table S2). Additionally, in the WQS regression model, after adjusting for serum Cu levels and for serum Cu levels plus demographic variables, the overall estimate of the model significantly increased, with the *p*-value decreasing from 0.0216 to 0.0002, indicating an enhanced significance of the model under different adjustments (Supplementary Table S3).

## 4 Discussion

Long-term hyperglycemia can lead to serious complications, including cardiovascular diseases, diabetic neuropathy, nephropathy, and retinopathy (19–23). These conditions significantly increase morbidity and mortality among diabetic patients. Recent studies have also indicated that heavy metals are key factors in the pathogenesis of several diseases, including obesity, metabolic syndrome, and hypertension (24–26). Environmental pollution caused by heavy metals has become an ongoing concern worldwide (27, 28). In recent years, the impact of exposure to individual heavy metals on blood glucose levels has garnered widespread attention. These metals, by interfering with the body’s normal metabolic functions, may increase the risk of diabetes. Research covering various heavy metals, including manganese (29), nickel (30), mercury (31), cadmium (32), and lead (33), has indicated that they disrupt glucose metabolism and insulin sensitivity through different mechanisms, thereby affecting blood glucose levels. However, all of the above studies did not include copper exposure, so it remains uncertain whether copper exposure is associated with abnormal changes in blood glucose in heavy metal mixtures.

In this study, we investigated the serum copper levels, inflammatory markers, and fasting blood glucose levels among 2,318 participants, uncovering a range of meaningful results. The average serum Cu level was found to be 119.50 μg/dL, indicating moderate variability among individuals. Through PCA, we identified that WBC, Lym, Mono, and Nsg as inflammatory markers occupied significant positions in the variability of the data. Mediation analysis further revealed the important mediating roles of WBC, Lym, Mono, and Nsg in the relationship between serum Cu levels and BG, with WBC showing the highest mediation proportion at 39.78%, and Nsg showing the lowest at 20.82%. During MLRA, we observed a positive correlation between WBC and serum Cu levels, while Mono showed a negative correlation with serum Cu levels. Additionally, using the WQS regression model, we explored the cumulative impact of inflammatory factors on BG and found a significant effect of the combination of inflammatory markers on BG levels. Sensitivity analysis confirmed the robustness of these findings, with the estimates of mediation effects and the WQS regression model being strengthened after adjusting for demographic variables.

Accumulating evidence supports the role of inflammation in the abnormal changes in blood glucose (34). Lin et al. (35) revealed the relationship between excessive intake of sugary drinks and abdominal obesity with diabetes and the elevation of C-reactive protein (CRP) levels, emphasizing the positive correlation between sugar intake and CRP levels in adults with prediabetes. Moreover, Mi et al. (36) found that the Dietary Inflammatory Index (DII) is positively associated with insulin resistance in adults of normal and healthy weight, highlighting the potential value of anti-inflammatory diets in the prevention or management of insulin resistance. Furthermore, Yuan et al. (37) explored the relationship between the DII and long-term all-cause and cardiovascular mortality, pointing out that high DII scores are associated with an increased risk of long-term all-cause and cardiovascular mortality in patients with diabetes.

Given this common pathogenesis, it is reasonable to investigate whether Cu exposure leads to abnormal changes in BG through inflammation. In this study, we found that WBC, Lym, Mono, and Nsg were involved in the positive correlation between serum Cu concentration and changes in BG concentration, accounting for 39.78, 10.16, 33.37, and 20.82%, respectively, in the mediation analysis. Therefore, we hypothesized that excessive Cu exposure promotes inflammation and thus increases blood glucose concentration.

The results of our sensitivity analysis underscore the stability of our findings when considering the effects of serum copper levels and demographic variables. Notably, the negative correlation between Mono and serum Cu levels remained consistent across various model adjustments, highlighting the potential of Mono as a robust indicator of serum Cu levels. However, the association between WBC and serum Cu levels became non-significant after adjusting for demographic variables, suggesting the need to consider a broader range of demographic and biological factors when evaluating the relationship between biomarkers and environmental exposure (38, 39). Finally, the results of the WQS model further support the importance of the relationship between serum Cu levels and health outcomes under different adjustments.

Our study possesses several strengths. First, this research constitutes the inaugural investigation into the impact of Cu exposure on BG concentration via inflammatory factors. Second, we utilized a variety of statistical methodologies and adjusted for potential confounding variables to enhance the robustness and reliability of our findings. Third, all data were sourced from a large population database and adhered to stringent quality control measures. However, this study also has certain limitations. First, due to its cross-sectional design, we cannot ascertain the causality between copper exposure and alterations in blood glucose levels. Second, the NHANES database lacks data on uncontrollable factors, such as exposure to wastewater and cosmetics, which might affect the accuracy of our results. Third, not considering the cumulative amount of copper exposure could impact the outcomes.

As for the future direction and suggestion, it is necessary to conduct long-term longitudinal studies to determine the causal relationship between Cu exposure and BG levels, and explore its molecular mechanisms, particularly how Cu affects BG regulation through inflammatory pathways. Moreover, we should further investigate how environmental Cu exposure interacted with lifestyle factors such as diet and physical activity to collectively influenced the development of abnormal BG concentration. Most importantly, for populations living in areas of high heavy metal exposure, healthcare providers should consider regular testing for serum Cu and other relevant biomarkers as part of routine health screenings. In addition, specific nutritional and medical advice should be provided to these populations to reduce the potential health risks of heavy metal exposure.

## 5 Conclusion

This research with 2,318 NHANES participants conclusively demonstrated that serum Cu levels significantly influenced BG concentrations through specific inflammatory markers, suggesting reducing heavy metal exposure in the environment could lower the risk of developing diabetes. Mediation analyses revealed the impact of inflammatory markers on BG, emphasizing the role of public health measures to reduce heavy metal exposure and consider environmental factors in reducing diabetes.

## Data availability statement

The datasets presented in this study can be found in online repositories. The names of the repository/repositories and accession number(s) can be found at: https://wwwn.cdc.gov/nchs/nhanes/Default.aspx.

## Ethics statement

The studies involving humans were approved by NCHS Ethics Review Board Protocol #2011–17. The studies were conducted in accordance with the local legislation and institutional requirements. Written informed consent for participation was not required from the participants or the participants’ legal guardians/next of kin in accordance with the national legislation and institutional requirements.

## Author contributions

ZC: Visualization, Writing – original draft. YK: Formal analysis, Project administration, Writing – original draft, Writing – review & editing. WY: Methodology, Project administration, Software, Writing – review & editing. HX: Investigation, Resources, Writing – review & editing. DT: Investigation, Methodology, Supervision, Writing – review & editing. YZ: Conceptualization, Investigation, Methodology, Writing – review & editing.

## Footnotes

^1^ https://wwwn.cdc.gov/nchs/nhanes/
