# Supplementary material for: Association between serum copper and blood glucose: a mediation analysis of inflammation indicators in the NHANES (2011–2016)
Source: Front Public Health. 2024 May 24;12:1401347. doi: 10.3389/fpubh.2024.1401347 (PMC11157037; doi:10.3389/fpubh.2024.1401347)
Supplement: Supplementary file 1 [file Data_Sheet_1.docx]

Table S1 The mediating effects of the relationship between serum Cu and BG (Adjusted for covariates)

| Model I* | Indierct Effects β (95% CI) | | | Dierct Effects β (95% CI) | | | Total Effects β (95% CI) | | | Mediated Proportion | P-value |
| --- | --- | --- | --- | --- | --- | --- | --- | --- | --- | --- | --- |
|  | Estimate | CI Lower | CI Upper | Estimate | CI Lower | CI Upper | Estimate | CI Lower | CI Upper |  |  |
| WBC | 0.08526 | 0.0281 | 0.1425 | 0.0158 | 0.0050 | 0.0267 | 0.1011 | 0.0447 | 0.1575 | 0.1566 | 0.0042 |
| LymPCT | 0.10188 | 0.0454 | 0.1584 | 0.0006 | -0.0027 | 0.0039 | 0.1025 | 0.0461 | 0.1589 | 0.0059 | 0.7203 |
| MonoPCT | 0.09423 | 0.0376 | 0.1509 | 0.0229 | 0.0124 | 0.0334 | 0.1171 | 0.0608 | 0.1735 | 0.1954 | 0.0000 |
| NsgPCT | 0.09768 | 0.0410 | 0.1544 | 0.0049 | -0.0017 | 0.0114 | 0.1026 | 0.0462 | 0.1590 | 0.0476 | 0.1430 |

*Note:*

*LymPCT = Lym percentage, MonoPCT = Mono percentage, NsgPCT = Nsg percentage*

*Model I adjusted for serum Cu concentration.*

Table S2 Results of MLRA (Adjusted for covariates)

| Variable | Model I | | | | Model II | | | |
| --- | --- | --- | --- | --- | --- | --- | --- | --- |
|  | estimate | Lower CI | Upper CI | p.value | estimate | Lower CI | Upper CI | p.value |
| WBC | 1.031 | 0.172 | 1.890 | 0.019 | 0.832 | -0.073 | 1.737 | 0.072 |
| LymPCT | -0.569 | -1.266 | 0.128 | 0.109 | 0.060 | -0.693 | 0.813 | 0.876 |
| MonoPCT | -1.574 | -2.599 | -0.550 | 0.003 | -1.852 | -2.941 | -0.763 | 0.001 |
| NsgPCT | -0.453 | -1.121 | 0.215 | 0.184 | -0.050 | -0.770 | 0.669 | 0.891 |

*Note:*

*LymPCT = Lym percentage, MonoPCT = Mono percentage, NsgPCT = Nsg percentage*

*Model I adjusted for serum Cu concentration. Model II adjusted for serum Cu concentration and demographic variables.*

Table S3 Results of WQS regression (Adjusted for covariates)

|  | Model I | Model II |
| --- | --- | --- |
| Estimate | 7.7174 | 7.0833 |
| P - value | 0.0045 | 0.0002 |
| WBC | 0.3563 | 0.6568 |
| LymPCT | 0.1892 | 0.1389 |
| MonoPCT | 0.0928 | 0.0236 |
| NsgPCT | 0.3618 | 0.1807 |

*Note: LymPCT = Lym percentage, MonoPCT = Mono percentage, NsgPCT = Nsg percentage*

*Model I adjusted for serum Cu concentration. Model II adjusted for serum Cu concentration and demographic variables.*
